# Association of Web-Based Physical Education With Mental Health of College Students in Wuhan During the COVID-19 Outbreak: Cross-Sectional Survey Study

**DOI:** 10.2196/21301

**Published:** 2020-10-05

**Authors:** Cheng-Hu Deng, Jing-Qiang Wang, Li-Ming Zhu, He-Wang Liu, Yu Guo, Xue-Hua Peng, Jian-Bo Shao, Wei Xia

**Affiliations:** 1 Department of Physical Education Wuhan University of Technology Wuhan China; 2 Department of Physical Education Hubei Business College Wuhan China; 3 Department of Physical Education Jianghan University Wuhan China; 4 Department of Physical Education Huazhong Agricultural University Wuhan China; 5 Department of Imaging Center Wuhan Children's Hospital (Wuhan Maternal and Child Healthcare Hospital) Tongji Medical College, Huazhong University of Science and Technology Wuhan China

**Keywords:** COVID-19, college students, mental status, physical education, young adults, web-based education, global health, web-based survey, physical activity, mental health

## Abstract

**Background:**

The COVID-19 outbreak has affected people’s health worldwide. For college students, web-based physical education is a challenge, as these course are normally offered outdoors.

**Objective:**

The aim of this study was to use data from a web-based survey to evaluate the relationship between the mental health status of college students and their sports-related lifestyles. Problems related to web-based physical education were also examined.

**Methods:**

A web-based survey was conducted by snowball sampling from May 8 to 11, 2020. Demographic data, mental health status, and sports-related lifestyles of college students in Wuhan as well as issues related to web-based physical education were collected. Mental health status was assessed by the Depression, Anxiety, and Stress Scale (DASS-21).

**Results:**

The study included 1607 respondents from 267 cities. The average scores of the DASS-21 subscales (2.46 for depression, 1.48 for anxiety, and 2.59 for stress) were significantly lower in our study than in a previous study (*P*<.05). Lower DASS-21 scores were significantly correlated with regular exercise, maintaining exercise habits during the outbreak of COVID-19, exercising more than 1 to 2 times a week, exercise duration >1 hour, and >2000 pedometer steps (all *P*<.05). None of the three forms of web-based physical education was preferred by more than 50% of respondents. Frequent technical problems were confronted by 1087/1607 students (67.6%). Shape-up exercises (846/1607, 52.6%), a designed combination of exercises (710/1607, 44.2%), and Chinese kung fu (559/1607, 34.8%) were suggested sports for web-based physical education.

**Conclusions:**

Mental status was significantly correlated with regular exercise and sufficient exercise duration. Professional physical guidance is needed for college students in selected sports. Exercises not meeting students’ preferences, frequent technical problems, and the distant interaction involved in web-based physical education were the main problems that should be solved in future.

## Introduction

In December 2019, a novel coronavirus, SARS-CoV-2, was first recognized in Wuhan and then quickly spread worldwide, infecting millions of people [1]. Furthermore, uninfected people were also greatly affected and were required to adopt a totally different lifestyle because of quarantine in Wuhan [2]. Many basic necessities of living, as well as education, were transferred to the internet [3,4]. Web-based education was not new in many fields [5]; however, it is novel in sports education, which requires essential interaction between teachers and students.

Previous studies have stated that mental illness symptoms are common among university students according to the Depression, Anxiety, and Stress Scale (DASS-21) [6,7]. Dogra’s study [8] described that vigorous physical activity is associated with lower possibility of poor mental health and depressive symptoms. As physical education plays an important role in relieving pressure not only on the body but also on the mind [9], it is particularly essential during the pandemic [10]. According to a report by the United Nations Educational Scientific and Cultural Organization (UNESCO), more than 160 countries have shut down schools [11]; therefore, web-based physical education is an issue that most countries must face. It has been reported that SARS-CoV-2 will coexist with humans for a long time [12], which suggests that web-based physical education could last for a relatively long time in the future. A recent scientometric analysis reviewed research topics related to COVID-19; however, the impact of COVID-19 on physical education was not researched [13].

Since February, web-based physical education, which is the only choice in a quarantined city, has been adopted by many universities and colleges in Wuhan [14]. Whether the web-based physical education was efficient or a waste of time may be reflected by the mental and physical state of students. Several studies have reported the mental state of the general population, medical staff, or college students during the peak of the COVID-19 epidemic; these studies have proved the existence of anxiety, depression, and stress in different populations [15-18]. However, as far as we know, no report has evaluated the mental and physical state of students in universities and colleges in Wuhan after the implementation of web-based physical education.

As a newly developed mode of physical education, web-based physical education presents challenges and opportunities. Because of the characteristics of physical education, suitable sports should be chosen while considering the restrictions of sports fields and related equipment. Also, the performance of network platforms and teaching resources for web-based physical education remains a question. Furthermore, the extended effects of web-based physical education on family members when the activities are performed at home is a concern, which may be related to the similar atmosphere to class learning.

Therefore, the primary aim of our study is to describe the association between the mental health and physical activity of students in Wuhan universities and colleges after a 3-month web-based physical education program. The secondary aim of our study is to identify the problems that students encountered during the web-based study, and we hope that these problems can be solved by educators in the near future.

## Methods

### Study Design and Study Population

This cross-sectional study was conducted from May 8 to 11, 2020. A snowball strategy was employed to recruit students in Wuhan universities and colleges after a 3-month web-based physical education program.

### Procedure

To minimize face-to-face interactions as recommended by the Chinese government, an anonymous questionnaire was completed through a web-based survey platform (SurveyStar, Changsha Ranxing Science and Technology). The web-based survey was first disseminated by teachers to students through the educational platform, and the students were encouraged to spread it to other students. This study was approved by the institutional review board of Wuhan Children’s Hospital (WHCH 2020029). Informed consent was requested at the very beginning of this web-based survey, and informed consent was provided by all the respondents.

### Measurements and Outcomes

A structured questionnaire was employed to evaluate the mental health status and sports-related lifestyles of students in Wuhan universities and colleges as well as their issues related to web-based physical education. There were 45 items included in the questionnaire, and approximately 6 minutes were required to complete it.

Demographic data included gender, age, grade, residential location, and BMI, which was calculated as weight in kilograms divided by height in meters squared. The categorization of BMI was based on reference data for Chinese people (BMI＜18.5, 18.5≤BMI＜23, 23≤BMI＜26, 26≤BMI) [19]. The mental health status of students was evaluated by the DASS-21, which consists of depression, anxiety, and stress subscales [20]. Sports-related lifestyle variables included exercise habits, changes in weight, frequency and duration of exercise, preferred sports, pedometer steps, and access to sports facilities. The minimal amount of weight to indicate weight loss or weight increase was 3 kg. The average number of pedometer steps was calculated as the average number of steps taken per day during the past month according to a mobile phone app, WeChat (Tencent). There was no control for other physical activities or exercises performed in addition to web-based physical education. Variables for characteristics of web-based physical education included different exercise classes offered, frequency of web-based physical education, influence on other family members which may be related to the similar atmosphere to class learning, problems confronted, and suggested sports for web-based physical education.

In addition to the total score of the DASS-21 scale, the scores of the three subscales were calculated as follows [15]: the depression subscale consisted of questions 3, 5, 10, 13, 16, 17, and 21, classified into normal (0-9), mild depression (10-12), moderate depression (13-20), severe depression (21-27), and extremely severe depression (28-42); the anxiety subscale consisted of questions 2, 4, 7, 9, 15, 19, and 20, classified into normal (0-6), mild anxiety (7-9), moderate anxiety (10-14), severe anxiety (15-19), and extremely severe anxiety (20-42); the stress subscale consisted of questions 1, 6, 8, 11, 12, 14, and 18, classified into normal (0-10), mild stress (11-18), moderate stress (19-26), severe stress (27-34), and extremely severe stress (35-42).

### Statistical Analysis

Respondents who did not complete the questionnaire were not included in our study; we deleted the respondents with missing data. Descriptive statistics were employed for categorical variables, including demographic data, sports-related lifestyle, and issues related to web-based physical education. Each categorical variable was presented as the percentage of responses to the corresponding question, which was calculated by dividing the number of respondents per response by the number of total responses to the question. The scores of the DASS-21 scale and its subscales were expressed as mean (SD) as well as median (IQR). The Cronbach alpha values of the reliability of the DASS-21 and its subscales were calculated as a measure of internal consistency. We performed a descriptive comparison of the mean scores of the subscales of the DASS-21 between our study and Wang’s first study [15], which described the mental health state of the general population during the peak of the COVID-19 epidemic. Linear regressions were used to analyze the univariate associations between sports-related lifestyle variables and the DASS-21 scales. All the tests were two-tailed, with a significance level of *P*<.05. Statistical analysis was performed using SPSS 19.0 (IBM Corporation).

## Results

### Survey Respondents

A total of 1673 surveys were received; as 66 respondents did not complete the questionnaire, we included 1607 (96.1%) respondents from 267 cities in the study. Questionnaires were received on the first day (May 8) from 292 respondents, on the second day (May 9) from 1130 respondents, on the third day (May 10) from 130 respondents, and on the fourth day (May 11) from 55 respondents.

### Demographic Data

The demographic features of all the students in our study are shown in Table 1. Most respondents were male (1041/1607, 64.8%) and were aged 18 to 22 years (1573/1607, 97.9%). Most of the students were freshmen or sophomores (1524/1607, 94.8%), and urban areas (723, 45%) were the most common place of residence of the college students. The BMI values of most of the 1607 college students were in the normal range (969, 60.3%), while the BMI values of 638 respondents (39.7%) were out of the normal range.

**Table 1:** The demographic characteristics of the respondents (N=1607), n (%).

Demographic characteristic		Value
**Gender**		
	Male	1041 (64.8)
	Female	566 (35.2)
**Age (years)**		
	<18	20 (1.2)
	18-22	1573 (97.9)
	>22	14 (0.9)
**Grade**		
	Freshman	784 (48.8)
	Sophomore	740 (46.0)
	Junior	70 (4.4)
	Senior	13 (0.8)
**Place of residence**		
	Urban	723 (45.0)
	Rural-urban	431 (26.8)
	Rural	453 (28.2)
**BMI, kg/m^2^**		
	＜18.5	275 (17.1)
	18.5-22	969 (60.3)
	23-25	231 (14.4)
	≥26	132 (8.2)

### Mental Health Status

The respondents’ mental health status was measured by the DASS-21 scale. The mean (SD) of the total DASS-21 score was 6.52 (7.86). The mean (SD) values of the depression, anxiety, and stress subscales were 2.46 (3.02), 1.48 (2.35), and 2.59 (3.09), respectively. The median (IQR) of the total score on the DASS-21 was 4 (1-10). The median (IQR) values of the depression, anxiety, and stress subscales were 1 (0- 4), 0 (0-2), and 1 (0-4), respectively. The comparison between the mean (SD) values in our study and Wang’s study [15] is as follows: for the depression subscale, 2.46 (3.02) versus 6.25 (7.16); for the anxiety subscale, 1.48 (2.35) versus 6.16 (6.57); for the stress subscale, 2.59 (3.09) versus 7.76 (7.74). The classification of responses in the different groups for the depression, anxiety, and stress subscales are shown in Table 2. The Cronbach alpha value of the reliability of the DASS-21 was .94, and the Cronbach alpha values for the depression, anxiety, and stress subscales were .84, .85, and .86, separately.

**Table 2:** Classification of responses to the depression, anxiety, and stress subscales of the DASS-21 (N=1607), n (%).

DASS-21^a^ subscale	Normal	Mild	Moderate	Severe	Extremely severe
Depression	1551 (96.5)	36 (2.2)	19 (1.2)	1 (0.1)	0 (0.0)
Anxiety	1519 (94.5)	71 (4.4)	14 (0.9)	2 (0.1)	1 (0.1)
Stress	1574 (97.9)	30 (1.9)	3 (0.2)	0 (0.0)	0 (0.0)

^a^DASS-21: Depression, Anxiety, and Stress Scale.

### Sports-Related Lifestyle and Exercise Status

The sports-related lifestyle variables of the college students who responded to the survey are shown in Table 3. Although most of the 1607 students (1088, 67.7%) exercised regularly, 1279 (79.6%) of the students were disturbed by the outbreak of COVID-19 and spent less time on sports (826, 51.4%) and/or gained weight (592, 36.8%). Exercising <3 times a week was observed in 1010/1607 students (62.9%), and <2000 average pedometer steps were observed in 1155/1607 students (71.9%). Restrictions on access to sports facilities were experienced by 1198/1607 students (74.5%).

**Table 3:** Sports-related lifestyle variables of the survey respondents after the outbreak of COVID-19 (N=1607), n (%).

Sports-related lifestyle variable		Value
**Regular exercise^a^**		
	Yes	1088 (67.7)
	No	519 (32.3)
**Negative influence of COVID-19 on exercise habits**		
	No, exercise habits were maintained	328 (20.4)
	Yes, but only slightly	889 (55.3)
	Yes, it has a great impact on my exercise habits	390 (24.3)
**Time spent on sports after the outbreak of COVID-19^b^**		
	Less	826 (51.4)
	Same	460 (28.6)
	More	321 (20.0)
**Weight change after the outbreak of COVID-19^c^**		
	Less	321 (20.0)
	Same	694 (43.2)
	More	592 (36.8)
**Frequency of exercise**		
	Occasionally or never	549 (34.2)
	1 to 2 times per week	461 (28.7)
	≥3 times per week	377 (23.4)
	Every day	220 (13.7)
**Average duration of exercise performed in a week (hours)**		
	<1	1253 (78.0)
	>1	354 (22.0)
**Favorite sport after the outbreak of COVID-19**		
	High-intensity interval training	119 (7.4)
	Shape-up exercises	200 (12.4)
	Strength training	312 (19.4)
	Ball game	288 (17.9)
	Walking	665 (41.4)
	Body combat	23 (1.4)
**Average pedometer steps^d^**		
	0-500	413 (25.7)
	501-2000	742 (46.2)
	2001-4000	224 (13.9)
	>4000	228 (14.2)
**Restricted access to sports facilities**		
	Yes	1198 (74.5)
	No	409 (25.5)

^a^Exercising regularly was defined as ≥3 times a week and ≥60 minutes each time.

^b^Time spent on sports was defined as time spent on all types of sports, including web-based physical education.

^c^The minimum amount of weight to indicate weight loss or weight increase was 3 kilograms.

^d^The average number of pedometer steps was calculated as the average number of steps taken per day during the past month according to the WeChat mobile phone app.

The differences in the association between sports-related lifestyle variables and the scores of the DASS-21 and its subscales are represented in Table 4. The respondents who exercised regularly had lower scores on the DASS-21 and all its subscales (for depression, B=–1.257, *t*=–7.962, *P*<.001; for anxiety, B=–0.700, *t*=–5.636, *P*<.001; for stress, B=–1.013, *t*=–6.211, *P*<.001; for total score, B=–2.969, *t*=–7.197, *P*<.001), as well as the respondents who maintained their exercise habits during the outbreak (for depression, B=–2.017, *t*=–9.171, *P*<.001; for anxiety, B=–1.211, *t*=–6.988, *P*<.001; for stress, B=–2.198, t=–9.788, *P*<.001; for total score, B=–5.427, *t*=–9.491, *P*<.001) or were influenced little by the outbreak (for depression, B=–1.301, *t*=–7.299, *P*<.001; for anxiety, B=–0.783; *t*=–5.572, *P*<.001; for stress, B=–1.446, *t*=–7.941, *P*<.001; for total score, B=–3.530, *t*=–7.616; *P*<.001). The respondents who exercised more than 1 to 2 times a week demonstrated significantly lower scores on the DASS-21 and all its subscales compared to respondents who exercised occasionally, with all *P*<.05. The respondents who exercised >1 hour had lower total scores (B=–1.350, *t*=–2.861, *P*=.004) and lower scores on the depression (B=–0.588, *t*=–3.248, *P*=.001) and stress (B=–0.503, *t*=–2.708, *P*=.007) subscales of the DASS-21 compared to respondents who exercised <1 hour. Respondents with >2000 average pedometer steps had significantly lower scores on the DASS-21 and all its subscales compared to respondents with <599 average steps, with all *P*<.05.

### Issues of Web-Based Physical Education

Three main modes of web-based physical education were adopted by universities and colleges in Wuhan. Interaction between teachers and students was the most common mode (1056/1607, 65.7%). Moreover, this was the only mode in which students could interact with the teacher; the other two modes involved unilateral teaching. Web-based physical education was accessed once per week or less by 1256/1607 students (78.2%). Surprisingly, the family members of 728/1607 students (45.3%) were motivated to exercise because of web-based physical education. Many problems arose during web-based physical education, which were confronted by 1087/1607 students (67.6%). Considering convenience and availability, the respondents suggested that shape-up exercises, designed combinations of exercise by teachers for a specific purpose, and Chinese kung fu were suitable sports for web-based physical education. Here, the designed combinations of exercise were combinations of various physical education exercises that were designed by teachers, such as a combination of shape-up exercises and Chinese kung fu. Detailed information is summarized in Table 5.

**Table 4:** Associations between sports-related lifestyle variables and scores on the DASS-21 and its subscales (N=1607).

Sports-related lifestyle variable		Depression subscale score			Anxiety subscale score			Stress subscale score				Total DASS-21^a^ score		
		B	*t*	*P* value	B	*t*	*P* value	B	*t*		*P* value	B	*t*	*P* value
**Exercise regularly^b^**														
	Yes	–.195	–7.962	<.001	–.139	–5.636	<.001	–.153		–6.211	<.001	–.177	–7.197	<.001
	No	Ref.^c^	N/A^d^	N/A	Ref.	N/A	N/A	Ref.		N/A	N/A	Ref.	N/A	N/A
**Negative influence of COVID–19 on exercise habits**														
	No, exercise habits were maintained	–.270	–9.171	<.001	–.208	–6.988	<.001	–.287		–9.788	<.001	–.279	–9.491	<.001
	Yes, but only slightly	–.215	–7.299	<.001	–.166	–5.572	<.001	–.233		–7.941	<.001	–.223	–7.616	<.001
	Yes, it has a great impact on my exercise habits	Ref.	N/A	N/A	Ref.	N/A	N/A	Ref.		N/A	N/A	Ref.	N/A	N/A
**Time spent on sports after the outbreak of COVID–19^e^**														
	More	–.075	–2.854	.004	–.037	–1.398	.16	–.046		–1.750	.08	–.058	–2.202	.03
	Same	–.102	–3.896	<.001	–.043	–1.645	.10	–.100		–3.807	<.001	–.091	–3.485	.001
	Less	Ref.	N/A	N/A	Ref.	N/A	N/A	Ref.		N/A	N/A	Ref.	N/A	N/A
**Weight change after the outbreak of COVID–1^f^**														
	Less	.018	0.633	.53	.012	0.442	.66	–.009		–0.322	.75	.007	0.249	.80
	Same	–.035	–1.275	.20	–.036	–1.300	.20	–.081		–2.938	.003	–.056	–2.034	.04
	More	Ref.	N/A	N/A	Ref.	N/A	N/A	Ref.		N/A	N/A	Ref.	N/A	N/A
**Frequency of exercise**														
	Every day	–.109	–3.998	<.001	–.095	–3.477	.001	–.086		–3.155	.002	–.104	–3.820	<.001
	≥3 times per week	–.146	–5.212	<.001	–.096	–3.387	.001	–.122		–4.337	<.001	–.133	–4.724	<.001
	1 to 2 times per week	–.112	–3.946	<.001	–.089	–3.124	.002	–.084		–2.949	.003	–.102	–3.613	<.001
	Occasionally or never	Ref.	N/A	N/A	Ref.	N/A	N/A	Ref.		N/A	N/A	Ref.	N/A	N/A
**Average duration of exercise performed in a week**														
	>1 hour	–.081	–3.248	.001	–.046	–1.833	.07	–.067		–2.708	.007	–.071	–2.861	.004
	<1 hour	Ref.	N/A	N/A	Ref.	N/A	N/A	Ref.		N/A	N/A	Ref.	N/A	N/A
**Average pedometer steps^g^**														
	>4000	–.123	–4.283	<.001	–.100	–3.487	.001	–.115		–4.026	<.001	–.122	–4.275	<.001
	2001-4000	–.103	–3.620	<.001	–.062	–2.175	.03	–.085		–2.971	.003	–.092	–3.212	.001
	501-2000	–.078	–2.566	.01	–.044	–1.443	.15	–.063		–2.078	.04	–.068	–2.236	.03
	0-500	Ref.	N/A	N/A	Ref.	N/A	N/A	Ref.		N/A	N/A	Ref.	N/A	N/A
**Restricted access to sports facilities**														
	No	–.040	–1.602	.11	–.027	–1.065	.29	–.084		–3.368	.001	–.056	–2.258	.02
	Yes	Ref.	N/A	N/A	Ref.	N/A	N/A	Ref.		N/A	N/A	Ref.	N/A	N/A

^a^DASS-21: Depression, Anxiety, and Stress Scale.

^b^Exercising regularly was defined as ≥3 times a week and ≥60 minutes each time.

^c^Ref.: reference.

^d^N/A: not applicable.

^e^Time spent on sports was defined as time spent on all types of sports, including web-based physical education.

^f^The minimum amount of weight to indicate weight loss or weight increase was 3 kilograms.

^g^The average number of pedometer steps was calculated as the average number of steps taken per day during the past month according to the WeChat mobile phone app.

**Table 5:** The conditions and problems of web-based physical education (N=1607), n (%).

Conditions and problems of web-based physical education		Value
**Participation in web-based physical education**		
	Watching recorded video	841 (52.3)
	Watching real-time video	820 (51.0)
	Communicating with a teacher on an education platform	1056 (65.7)
**Preferred type of web-based physical education**		
	Watching recorded video	497 (30.9)
	Watching real-time video	536 (33.4)
	Communicating with a teacher on an education platform	574 (35.7)
**Frequency of participation in web-based physical education**		
	Once every two weeks	25 (1.6)
	Once per week	1231 (76.6)
	Twice per week	351 (21.8)
**Has web-based physical education motivated other family members to exercise?**		
	Yes	728 (45.3)
	No	879 (54.7)
**Problems confronted during web-based physical education**		
	Network instability	752 (46.8)
	Lack of familiarity with software	384 (23.9)
	No interaction with teacher	481 (29.9)
	Lack of self-control	608 (37.8)
	Inability to keep up with the lesson	73 (4.5)
**Frequency of technical problems (network, software, platform)**		
	Every class	142 (8.8)
	>4 times per month	270 (16.8)
	≤4 times per month	675 (42.0)
	Never	520 (32.4)
**Would you like to continue to participate in web-based physical education?**		
	Yes	760 (47.3)
	No	847 (52.7)
**Suitable sports suggested for web-based physical education**		
	Shape-up exercise	846 (52.6)
	Designed combination of exercises	710 (44.2)
	Chinese kung fu	559 (34.8)
	Rhythmic sport	459 (28.6)
	Table tennis	364 (22.7)

## Discussion

### Principal Findings

The main purposes of our study were to determine the mental status of college students using the DASS-21 after 3 months of web-based physical education and to evaluate the relationship of the students’ mental health status with their sports-related lifestyle. A web-based survey was employed to collect related information. The results showed that the average scores on the DASS-21 subscales were significantly lower than in a previous study. Lower DASS-21 scores were significantly correlated with positive sports-related lifestyle. Furthermore, web-based physical education was unsatisfactory due to several issues related to technology and content.

#### Relationship Between Mental Health Status and Sports-Related Lifestyle

Previous studies have proven that the general population, including college students, suffered numerous negative effects induced by the outbreak of COVID-19 [21,22]. According to the DASS-21, depression, anxiety, and stress could be observed in various populations during the outbreak of COVID-19 in China, which lasted for at least 4 weeks [15]; these populations included the general population [21], general workforce [23], psychiatric patients [24], and health care professionals [25]. All groups in the general population were required to change and rebuild their lifestyles. For college students, in addition to the changes in their ordinary lifestyles, a brand new education style was rapidly established. Physical education, typically an outdoor course, was required to be conducted on the internet. Whether physical education is essential during the COVID-19 epidemic and how to suitably provide it are issues that most countries will be facing for a long period of time.

In Wang’s study [15], which reported a higher psychological impact of COVID-19 in respondents aged 12-21.4 years, it was suggested that respondents in this age group might be affected by prolonged school closure and require web-based education support. Comparing our study with Wang’s study, we observed lower scores on the DASS-21 subscales, which were obtained 3 months after web-based physical education was established. The difference between our study and the previous study may result from the restoration of web-based education support, of which physical education was an important part. To demonstrate the importance of exercise in our study, linear regression was employed to analyze the correlation between the DASS-21 scores and sports-related lifestyle variables. Unsurprisingly, lower scores on the DASS-21 and its subscales were observed in respondents who exercised regularly and maintained their exercise habits during the outbreak of COVID-19. The respondents who exercised more than 1 to 2 times a week, had an exercise duration >1 hour, and had >2000 average pedometer steps had significantly lower scores on the DASS-21 and all its subscales compared to other participants. These data suggest that exercise, especially regular exercise with sufficient duration, is related to a lower risk of mental disturbance, which is in accordance with a previous study [26]. It is surprising that although more than 70% respondents accumulated <2000 pedometer steps per day, the mean DASS-21 scores were low. We believe that the low number of steps per day may be related to the exercises chosen for the physical classes during COVID-19 confinement. Some exercises may not involve many steps, such as tai chi and shape-up exercises. According to a large cross-section study, which verified that all exercise types are significantly associated with lower mental health burden [27], the exercise itself mattered rather than the type. Strong evidence from a meta-analysis supported that exercise can protect populations from depression regardless of age and geographical region [28], which may also apply to the COVID-19 pandemic. Therefore, we suggest that a positive sports-related lifestyle is significantly associated with mental health during the confines of the COVID-19 pandemic.

#### Issues Related to Web-Based Physical Education

It is unfortunate that 1198/1607 respondents (74.5%) were restricted from using sports facilities, which may be an obstacle preventing them from exercising regularly. Moreover, the BMI of 638/1607 respondents (39.7%) was out of the normal range, which may be related to an unhealthy lifestyle [29]. These results suggest that the respondents were in need of professional guidance for physical education, considering the available facilities and equipment. Interestingly, 728/1607 respondents (45.3%) observed that other family members were motivated to exercise by web-based physical education. This may be related to the similar atmosphere to class learning, in which the other participating family members can be considered as classmates or companions. This also suggests that web-based physical education is not only a new learning style for college students but may also be a new lifestyle for the general population [30,31].

As stated above, effective web-based physical education is essential for lifestyle rebuilding not only for college students but also for the general population as part of behavior therapy [32], health education [33], and promotion by local health authorities [34]. However, as a totally new mode of physical education, web-based learning involved several issues that must be noted. As far as we know, only three types of web-based physical education were available to the college students, including watching recorded video, watching real-time video, and communicating with a teacher on an education platform. However, none of these types was preferred by more than 50% of respondents, and more than 50% of respondents did not want to experience physical education on the internet. However, in contrast, motivation of family members to exercise by web-based physical education was observed by nearly 50% of respondents. This suggests that web-based physical education is welcomed by the general population but cannot meet the requirements of college students. We believe that the dissatisfaction of college students may result from the comparison between web-based physical classes and face-to-face classes, whereas the general population would be more interested in trying this new style without having experienced face-to-face classes. Moreover, frequent technical problems were confronted by 1087/1607 respondents (67.6%), including network instability (752/1607, 46.8%) and unfamiliarity with software (384/1607, 23.9%), which further worsened the experience of the physical course. Furthermore, lack of interaction with the teacher (481/1607, 29.9%), lack of self-control (608/1607, 37.8%), and inability to keep up with the lesson (73/1607, 4.5%) could be observed in the respondents, which was frustrating for the college students. However, Soffer’s study [35] suggested that in many aspects of the examined effectiveness, web-based education was at least as effective as a face-to-face course [35]. We suppose that web-based physical education is promising if substantial improvements are made, such as technology support, optional exercises, and accommodation of students’ preferences. According to Chekroud’s study [27], all exercise types were significantly associated with lower mental health burden; numerous types of exercises could be chosen for web-based physical education. Considering the access to sports facilities and available sports equipment, only a few sports were suggested for web-based physical education, including shape-up exercises, a designed combination of exercises, Chinese kung fu, and rhythmic sport, and table tennis; meanwhile, ordinary physical courses could not be conducted properly, such as ball games and athletic events. Improving the physical fitness of college students with limited sports is the major issue of web-based physical education; to address this issue, we may learn from other courses and search for help from other fields, such as virtual reality [36].

### Limitations

There are several limitations of our study. First, due to anonymity and confidentiality requirements, a prospective study could not be performed through the web-based snowball sampling survey, and the respondents to the survey may not be a representative sample of Chinese students. Second, mental health was evaluated by the DASS-21 scale instead of by mental health professionals; floor effects could not be excluded, although both methods of evaluation are based on the respondents’ feelings and self-reporting. Third, due to the inherent nature of a cross-sectional study, we could only verify the association between sports-related lifestyle and mental health and could not verify the causal relationship. Finally, the assessment of sports-related lifestyle variables depends on non-standardized questions that have not been validated.

### Conclusions

The mental status of most of the college students in Wuhan who responded to our survey was normal. The mental status of the students was significantly correlated with regular exercise and sufficient exercise duration. Therefore, professional physical guidance is needed for college students as well as the general population. Considering the restrictions on sports facilities and equipment, selected sports were suggested for web-based physical education to improve physical fitness. However, web-based physical education is still far from satisfactory. Exercises not meeting students’ preferences, frequent technical problems, and distant interactions are the main problems that should be solved in future.

## Abbreviations

DASS-21: Depression, Anxiety, and Stress Scale
